# Experience of people living with leprosy at leprosy settlements in Nigeria

**DOI:** 10.1002/puh2.171

**Published:** 2024-04-14

**Authors:** Gabriel Ilerioluwa Oke, Ifeanyi Nsofor, Bashar Abubakar, Don Eliseo Lucero‐Prisno, Ademola Peter Sunday, Ernesto Oluwafemi Dibia, Emmanuel Ebuka Elebesunu, Obadiah Okpokpo, Odinaka Kingsley Obeta, Abdulhammed Opeyemi Babatunde, Adebowale Sylvester Adeyemi, Philip Adewale Adeoye, Edith Nnenna Utaka

**Affiliations:** ^1^ Masters of Global Health Delivery Program University of Global Health Equity Butaro Rwanda; ^2^ Behavioral Insights Lab Seattle Washington USA; ^3^ Nigeria Health Watch Abuja Nigeria; ^4^ Global Health Focus/London School of Hygiene & Tropical Medicine London London UK; ^5^ Department of Medical Laboratory Science Ladoke Akintola University of Technology Ogbomoso Oyo Nigeria; ^6^ Department of Biomedical Laboratory Science University of Ibadan Ibadan Oyo Nigeria; ^7^ Department of Medical Laboratory Sciences University of Nigeria, Enugu Campus Enugu Enugu Nigeria; ^8^ Department of Medical Laboratory Sciences Ambrose Alli University Ekpoma Ekpoma Edo Nigeria; ^9^ Department of Medical Laboratory Sciences University of Jos Jos Plateau Nigeria; ^10^ Department of Medicine and Surgery Faculty of Clinical Sciences College of Medicine University of Ibadan Ibadan Oyo Nigeria; ^11^ Arkland Health Limited Abuja Nigeria; ^12^ Department of Community Medicine Jos University Teaching Hospital Jos Plateau Nigeria; ^13^ Centre for Health Systems Support and Initiatives for Development Abuja Nigeria

**Keywords:** healthcare delivery, leprosy, leprosy control, neglected diseases, Nigeria

## Abstract

**Background:**

Although Nigeria achieved the national leprosy elimination target of less than 1/10,000 population in 1998, factors such as culture, behavioural patterns and social determinants, among others, continue to contribute to an increase in leprosy cases and a poor state of living for individuals with leprosy in Nigeria. This study delves into the experiences of individuals residing in leprosy settlements in Nigeria.

**Methods:**

This study employed a community‐based cross‐sectional design, utilizing a concurrent mixed‐methods approach for comprehensive data collection. Questionnaires, focus groups and interviews are conducted simultaneously. The research involves participants from seven leprosy communities across Nigeria's six geopolitical zones and Federal Capital Territory. Qualitative methods, including 14 focus group discussions and 6 key informant interviews, are complemented by quantitative questionnaires, engaging residents, leaders and nongovernmental organization (NGO) representatives. Respondents comprised 35 leprosy patients, 21 family members, 7 community leaders, 7 settlement officers and 2 organizational heads involved in leprosy control.

**Results:**

The results indicate significant access to healthcare (93.7%) and interest in self‐care practices (95.2%), with a considerable proportion (74.6%) receiving free healthcare. Interview data underscore the limited government support, with NGOs and partners assuming a more substantial role. Qualitative insights from persons living with leprosy highlight financial struggles, stigmatization and substandard living conditions in settlements, exacerbated by limited government funding. This reliance on private and NGOs is further compounded by declining funding, hindering individuals’ ability to start businesses and provide self‐care.

**Conclusion:**

This study underscores the pressing need for increased government support, funding and better living conditions for individuals affected by leprosy in Nigeria. It highlights the significance of education, awareness campaigns and human rights promotion to combat stigma and enhance the quality of life for those living with leprosy. Moreover, the study advocates for the reintegration of affected individuals into their communities to foster societal inclusion and well‐being.

## Graphical abstract

This study delves into the experiences of individuals residing in leprosy settlements in Nigeria. The study underscores the need for collaborative efforts between the Nigerian government and partners to enhance and sustain leprosy control programmes.

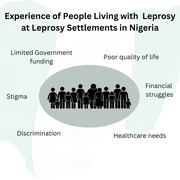

## INTRODUCTION

Leprosy remains a serious public health concern in Nigeria, with over 3500 people diagnosed each year and approximately 25% of these patients suffer from one form of disability or the other [1]. Before Nigeria's independence in 1960, Nigeria had a leprosy prevalence of more than 20% [2]. In conformity with existing global thinking and practice at the time, leprosy communities were developed to preserve public health and provide relief to men and women suffering from the disease.

Even though Nigeria achieved the national leprosy elimination target of less than 1/10,000 population in 1998 [2], some factors such as culture, behavioural patterns and social determinants, among others, are still contributing to more leprosy cases. The National Tuberculosis and Leprosy Control Programme (NTBLCP) started as a programme in 1989 [3]. Between 1991 and 2012, 111,788 leprosy patients were effectively treated with multidrug‐resistant treatment, and the country was also able to meet another target which was the WHO eradication target of fewer than one case per 10,000 people in 2000 [4]. With a case detection rate of less than 5% per 10,000 persons, Nigeria is currently classified as low endemic for leprosy; however, at the sub‐national level, there are areas of ‘high endemicity’ where leprosy prevalence remains at up to 1 instance for every 10,000 persons [2].

In 2019, 10% (20,205) of global leprosy cases were recorded in Africa with Nigeria and 12 other countries reporting 1000–10,000 cases [5]. Studies from high leprosy endemic countries have shown that leprosy shows marked uneven geographical distribution even within the smallest community groups such as villages and households [6]. This is one of the motivations for this research as people living with leprosy are mostly living in isolated communities outside main towns and cities.

Some other motivation for this study is the lack of publication and inadequate conversation about the level of care available for people living with leprosy in Nigeria. In Nigeria, we cannot compare the level of reporting tuberculosis (TB) to that of leprosy even by the NTBLCP. The NTBLCP is an integral part of the Nigerian Ministry of Health with the role of coordinating and implementing national strategies, policies and programmes for the control and management of TB, leprosy and Buruli ulcer in Nigeria [7]. Some of the programme's responsibilities encompass policy development, strategic planning, programme coordination, capacity building, monitoring and evaluation, advocacy and awareness, and research and innovation. Meanwhile, the level of stigma and disdain experienced by some people living with leprosy is so disheartening as leprosy is seen as the most terrible and despised disease in some areas of Nigeria [8].

The stigma has long‐term negative consequences for people with disabilities, including loss of employment, social ties and reputation, difficulties finding a life partner, divorce and discrimination [9]. As a result, leprosy is frequently referred to as a social killer [10]. The stigma associated with leprosy is sometimes more distressing than the disease itself [11]. Some leprosy patients have frequently described how they are denied some social and economic opportunities. Some are also rejected by family members and society. These events have repercussions on their psychological health. Some, because of stigma and disdain, therefore, postpone receiving treatment until difficulties appear [12].

Nigeria through the Federal Ministry of Health and the NTBLCP and partners has successfully treated over 33,000 leprosy patients across the country since 2009 till date; this has been made possible through funding from donors and nongovernmental organizations (NGOs) [13]. International Federation of Anti–Leprosy Associations (ILEP) is a consortium of organizations that provides relief and rehabilitation services to persons affected with leprosy and their efforts are complemented by efforts from other organizations outside ILEP. ILEP organizations include the Leprosy Mission, Nigeria (coverage is nationwide), the Damien Foundation (covers the South‐Western states and Kwara state with a coverage population of over 56 million people) and Deutsche Lepra‐ und Tuberkulosehilfe e.V. (DAHW) German Leprosy and TB Relief Association (area of coverage is South‐East and South–South, Nigeria) [14]. Nigeria has made some level of progress in the fight against leprosy. However, to record tangible progress, there is a need for the Nigerian government to increase its commitment to leprosy control and elimination by working alongside partners to ensure effective and sustainable leprosy control in line with the 2021–2025 global leprosy strategy [13]. This study explored the experiences of people living with leprosy, the experiences of healthcare workers and the conditions of living in the leprosy settlements.

## METHODS

### Study design

This study adopted a community‐based cross‐sectional design, utilizing a concurrent mixed‐methods approach for comprehensive data collection. The methodology integrated both quantitative cross‐sectional study and qualitative in‐depth interviews, employing a concurrent triangulation framework. This ensures a thorough exploration of the experiences of individuals living with leprosy in selected settlements. Although quantitative and qualitative data were collected simultaneously, they were analysed separately to maintain methodological rigor.

### Sampling strategy

The study encompassed 64 leprosy settlements in Nigeria, with 7 representing diverse geo‐political zones (6) and Federal Capital Territory. A cluster sampling approach was employed, partitioning settlements into zones and randomly selecting one per zone.

Additionally, interviews were held with community leaders and designated individuals responsible for overseeing each of the seven leprosy settlements. This selection approach was deemed appropriate as all participants shared similar living environments and common experiences. The paramount objective was to gather diverse perspectives from within the same population, aligning with our qualitative research goals.

The two representatives of organizations interested in leprosy control were purposively selected based on their contributions to leprosy control within their respective regions. The agencies and organizations whose staff were interviewed include the Damien Foundation, the Leprosy Mission Nigeria (TLMN) and DAHW German leprosy and Tuberculosis Relief Association. They were interviewed virtually via Zoom, and the meetings were recorded with consent.

These approaches allowed for a diversity of perspectives while ensuring that the study was feasible within the available resources and time frame. It is important to note that our primary aim was to explore and understand the experiences and perceptions of individuals living with leprosy, their families and community leaders, rather than to make generalizable claims about the entire population.

### Study locations

There are 64 leprosy settlements in Nigeria, and this number is unstable as some of these settlements are no longer functioning. Seven leprosy settlements were selected through cluster sampling, including the only settlement in FCT, study locations: (i) South‐West; the Lepers’ Colony, Ogbomosho, Oyo state; (ii) South–South; The Lepers' Colony, Osiomo, Edo state; (iii) South‐East; Uzuakoli Leprosy Colony, Abia state; (iv) North‐Central; Chanchaga Leprosy Hospital, Niger state; (v) North‐East; RafinKada Leper Colony, Wukari, Taraba state; (vi) North‐West; Lepers’ Colony, KutareGusau in Zamfara state; (vii) Alheri community, Yangoji village, Kwali Area Council, FCT.

#### Selection of participants

The decision to include nine participants in the quantitative aspect was a pragmatic choice, aligning with the qualitative objectives of the study and being mindful of resource constraints. Employing a purposive sampling method, we briefed the officials responsible for the settlements on the study objectives and expressed our desire to include every member of the population at their settlements. They actively contributed to participant selection for both the quantitative and qualitative components of the study, focusing on individuals who could provide valuable insights. The only exclusion criterion was any prior lack of intelligibility or ability to hear or speak.

Random selection was conducted based on these biases and conditions. The research team operated under the guidance of the consensus reached by the government‐appointed individual in charge of the leprosy settlement and the community head representing the community. This collaborative approach facilitated the random selection of the nine participants for the quantitative study, adhering to the desired distribution of five persons living with leprosy and three family members. This strategic sampling method enabled a thorough exploration of the research goals without the necessity for a larger quantitative sample.

The nine participants selected for the quantitative component were chosen in light of the exploratory and qualitative nature of our research. Our primary goal was to uncover in‐depth insights and qualitative data rather than engage in inferential analysis or make population‐level conclusions. Consequently, the sample size was not driven by the need for representativeness but rather by the aim of providing a rich and diverse source of data to support the qualitative responses. On the qualitative front, we conducted one focus group discussion with eight male participants and another with eight female participants.

The emphasis on qualitative results within this study led to a deliberate adjustment in our approach. The research cohort comprises five persons living with leprosy, three family members, one community leader from each of the seven selected settlements and one leader in the settlements that were appointed by the government. One FGD with a male population and one with a female population were done in all selected settlements. This was thought to be appropriate since all persons lived in the same environment. Participants who were selected to be part of the quantitative were not part of the whole that was selected for the qualitative component of the study.

The choice of nine participants for the quantitative aspect aligns with the exploratory and qualitative nature of the research, aiming for in‐depth insights rather than representativeness. This study was designed to make exploratory and qualitative submissions, and no inferential analysis was intended. Quantitative data were used to describe and support qualitative responses, and not to conclude, so representativeness was not considered.

**Table:** 

Study location	Type of study	Study participants			
**Seven selected settlements**	**Qualitative(14 focus group discussions and 14 key informant interviews)**	One FGD with eight males	One FGD with eight females	One key informant interview with the community leader	One key informant interview with the person assigned to the centre by the government
	**Quantitative using questionnaires**	Five persons living with leprosy	Three family members	One community leader	
**Three non‐governmental organizations**	**Qualitative(six Key informant interviews)**	Two representatives of the Damien Foundation	Two representatives of The Leprosy Mission Nigeria (TLMN)	Two representatives each of the DAHW German Leprosy and Tuberculosis Relief Association	

*Note*: Summary of study participants.

### Data collection

Quantitative data stem from structured questionnaires, whereas qualitative dimensions arise from Key informant interviews and focus group discussions. Questionnaires assess health centres, water supply, sanitation, hygiene, human resources and healthcare resources. The focus group discussions and interviews with settlement leaders include descriptions of the quality of life of people living in leprosy communities and the amenities available.

The quantitative questionnaire provides baseline data, complemented by focus groups and interviews delving deeper into participants’ experiences. Key informant interviews were conducted with settlement heads, both community‐appointed and government‐appointed. Questionnaires were administered to five persons living with leprosy, three family members who were randomly selected, and a community leader. The sample size was limited to nine respondents for the quantitative aspect. Moreover, groups of eight males and eight females each were also engaged in a focus group discussion, and the community leaders and persons appointed to be in charge were interviewed in the seven leprosy settlements.

The FGD sessions enabled exploratory and confirmatory questions to be asked to achieve a deeper understanding of their interests and needs. Focus groups were constructed in ways that will not hamper the discussion of sensitive topics due to differences in occupation, lifestyle, roles and status in the community. This allowed participants to discuss topical issues in detail and explore and clarify their points of view, thus enhancing in‐depth discussions. The questionnaire and interview questions were translated into local languages before data collection. Two data collectors who understand local languages accompanied research assistants to the selected leprosy settlements. This study does not measure the expertise of workers or professionals but explores the experiences of persons living with leprosy.

### Pretesting and calibration

After conducting a literature review, the questionnaire and interview questions underwent an iterative refinement process to optimize the protocols. Subsequently, these refined tools were pilot‐tested with four participants and settlement heads in Abuja. Valuable insights from representatives of TLMN were incorporated to enhance and consolidate the instruments for data collection.

#### Data analysis

NVIVO and IBM SPSS 25 were used for qualitative analysis and quantitative analysis of responses, respectively. Quantitative data were entered into Microsoft Excel 2010 from where the dataset was imported into SPSS. Data were subsequently cleaned, coded and analysed. Quantitative data were presented as frequencies and proportions. For the qualitative data, transcripts and field notes were analysed using thematic analysis to provide an accurate reflection of participants’ ideas. NVIVO 10 software was used for the systematic data coding to generate recurring themes by two data analysts. Another member of the team subsequently triangulated 10% of the transcripts to improve validity and draw up more perspectives which were compared with those generated by NVIVO analysis. This is necessary to reduce bias and revise the themes that might have occurred due to discrepancies and unexpected findings [15]. The team subsequently reviewed the generated themes to ensure that they reflected respondents’ ideas as opposed to the likelihood of bias often associated with a single analyst.

The outcomes of the study are listed below, and no cause‐effect relationship was measured. Moreover, outcomes were not measured but only described based on responses from respondents.

- Experiences of persons living with and affected by leprosy in Nigeria
- Challenges faced in leprosy settlements in Nigeria
- Experience of medical professionals working at leprosy settlements
- Reasons for abysmal financing and reporting measures of health financing
- State of health at leprosy settlements
- State of the settlements and recommendations for improvement
- Recommendations for leprosy control in Nigeria.

### How we mitigated biases

To address potential biases, our study employed a mixed‐methods design with concurrent triangulation, enhancing the robustness of our findings. Rigorous sampling involved cluster sampling with a collaborative approach, balancing the need for representation in the qualitative phase. Pragmatically, the quantitative sample size was limited to nine, aligning with the qualitative research goals. Pretesting and calibration were conducted iteratively, involving key stakeholders to refine instruments. During data analysis, NVIVO and SPSS facilitated thorough examination, and the team adopted measures for inter‐coder reliability in thematic analysis. Acknowledging limitations, virtual interviews were conducted, introducing potential biases, which were mitigated through transparency, reflexivity and triangulation.

#### RESULTS

As expressed in Table 1, the majority (66.7%) of the respondents were males; mostly (87.2%) older than 25 years (adults); with more than one third (38.1%) with no formal education. More than half are married (57.1%); half (50.8%) are traders and farmers but about a quarter (23.8%) are unemployed. Almost half (47.9%) of the residents have lived in the settlement for more than 20 years; with most (74.6%) of the residents earning less than the minimum wage (N 30,000).

**TABLE 1:** Socio‐demographic of respondents living in leprosy settlements in this study.

Variable	Frequency	(%)
Total	63	100
Study participants (*n* = 63)		
People living with leprosy	28	44.4
Family	21	33.3
Community leader	7	11.1
In charge	7	11.1
Sex (*n* = 63)		
Male	42	66.7
Female	21	33.3
Age years		
≤20	4	6.3
21–25	4	6.3
26–30	5	7.9
31–35	7	11.1
36–40	7	11.1
41–45	7	1.1
46–50	5	7.9
51–55	10	15.9
56–60	8	12.7
≥61	6	9.5
Education		
No formal education	24	38.1
Secondary education (complete)	12	19.0
Primary education (complete)	11	17.5
Primary education (incomplete)	5	7.9
Vocational studies	3	4.8
Secondary education (incomplete)	3	4.8
University/Polytechnic (Higher National Diploma) complete	2	3.2
NCE/Polytechnic (Ordinary National Diploma) incomplete	2	3.2
University/Polytechnic (Higher National Diploma) incomplete	1	1.6
Marital status		
Married	36	57.1
Single	14	22.2
Separated	7	11.1
Widowed	6	9.5
Occupation		
Farmer	18	28.6
Unemployed	15	23.8
Trader	14	22.2
Labourer	7	11.1
Civil servant	4	6.3
Carpenter	1	1.6
Welder	1	1.6
Nomadic Fulani	1	1.6
Retired civil servant	1	1.6
Student	1	1.6
Years lived in the community		
>20	35	47.9
≤10	17	27.0
11–20	11	17.5
Monthly income		
N < 30,000	47	74.6
N ≥ 30,000	8	12.7
No income	8	12.7

As indicated in Table 2, the majority (76.2%) of the respondents do not know the cause of Leprosy. Less than a quarter (22.2%) knows how it is transmitted. Half (52.4%) know that it is difficult to treat; with most (69.8%) of the respondents knowing the signs and symptoms of the disease. About two thirds (69.8% and 68.3%, respectively) indicated that leprosy causes shame, disdain or embarrassment, and leprosy is a source of problems in intimate relationships. The vast majority of respondents (87.3%) perceived negative attitudes towards trading with individuals living with leprosy, whereas a significant portion (61.9%) expressed concerns about the impact of leprosy on employment opportunities.

**TABLE 2:** Leprosy knowledge and perception of respondents living in leprosy settlements in Nigeria.

*n* = 63		
Variable	Frequency	(%)
Total	63	100
**Knowledge of the cause of leprosy**		
Yes	9	14.3
No	48	76.2
I don't know	6	9.5
**If yes, choose one**		
Bacteria	7	11.1
Diabolic	1	1.6
**Knowledge of the mode of transmission of leprosy**		
Yes	14	22.2
No	42	66.7
I don't know	7	11.1
**Leprosy is difficult to treat**		
Yes	33	52.4
No	22	34.9
I don't know	6	9.5
**Knowledge of the signs and symptoms of leprosy**		
Yes	44	69.8
No	18	28.6
I don't know	1	1.6
**Leprosy causes shame, disdain, or embarrassment**		
Yes	39	69.8
No	19	30.2
Don't know	1	1.6
Possibly	4	6.3
**Leprosy is a problem for a person to be in an intimate relationship**		
Yes	43	68.3
No	14	22.2
Don't know	1	1.6
Possibly	5	7.9
**People dislike trading with a person affected by leprosy**		
Yes	55	87.3
No	4	6.3
Don't know	2	3.2
Possibly	2	3.2
**Leprosy causes difficulty for a person to find work**		
Yes	39	61.9
No	11	17.5
Don't know	13	20.6

According to Table 3, the majority (93.7%) of the respondents have access to healthcare in the health centres. About a third (31.7% and 36.5%, respectively) reported leprosy symptoms at a health facility only when home treatment did not work. The majority (95.2%) are interested in learning self‐care practices for skin‐related neglected diseases. About three fourths (74.6% and 74.6%, respectively) receive free healthcare and accent that persons living with leprosy are admitted into the same ward as other patients. The majority (95.2%) think that the government should take special care of persons living with leprosy in Taraba State.

**TABLE 3:** Health services for people living with leprosy in leprosy settlements in Nigeria.

Variable	Frequency	(%)
Total	63	100
**Access healthcare in the health centre**		
Yes	59	93.7
No	4	6.3
**At what point will you go to a health facility when you develop a skin condition**		
When the problem has lasted for more than 1 week	5	7.9
When the problem has lasted for 2–4 weeks	10	15.9
When home treatments do not work	20	31.7
As soon as I realized that skin‐related neglected diseases have started	23	36.5
When the problem lasts for a year or two	2	3.2
I will not go to the healthcare facility to seek help	3	4.8
**Interested in learning self‐care practices for skin‐related neglected diseases**		
Yes	60	95.2
No	2	3.2
I don't know	1	1.6
**Access health care for free**		
Yes	47	74.6
No	16	25.4
**Persons living with leprosy are admitted into the same ward as other patients**		
Yes	47	74.6
No	16	25.4
**Government should take special care of persons living with leprosy**		
Yes	60	95.2
No	1	1.6
I don't know	2	3.2

### Qualitative data

#### Theme 1: the experiences of persons living with and affected by leprosy in Nigeria

Persons living with leprosy shared their experiences during the discussions, which, along with the perspectives of individuals in charge of the settlements and representatives of NGOs, were summarized into the following sub‐themes:

1. Available funding provisions:

Within leprosy settlements, there is a rising apprehension regarding funding, marked by uncertainty regarding the amount or assurance of financial support. The interview data substantiate the observation that there is limited funding support from the government and other implementing partners in Nigeria for individuals living with leprosy.

*I'm not sure but there's an allocation for drugs – a person living with leprosy in Taraba State*

*I have no idea except for the 5000 Naira monthly payment from the Local Government council* – a person living with leprosy in Taraba State from Oyo State

*I can't say exactly how much the government has budgeted but I can say that there is a fund set aside for People living with leprosy in Taraba State. There's a body for TB and leprosy control (NTBLCP) and the body runs a budget but it's minimal because most people focus on TB but yes there's funding but I can't say how much is budgeted exactly* – Staff of Damien Foundation

*There's no specific funding targeted at providing healthcare or a health insurance scheme. Even referral centres … suffer neglect, although leprosy treatment is free. Medications are provided by WHO and it's supplied to the Healthcare centres. Prednisolone is not free so the leprosy mission steps in to provide these drugs for the hospitals* – Staff of TLMN

It seems that most of the funding available at leprosy settlements is made available by NGOs, private bodies and not the government. *We receive support, the support comes from philanthropists, organizations like NNPC, and well‐meaning individuals. We also contribute money amongst ourselves to take care of minor things. –* Person living with leprosy in Taraba State Edo State

2. Condition of persons living with leprosy at the leprosy settlements and the state of the settlements

Living with leprosy was widely regarded as a difficult condition by the respondents. The leprosy settlements lack basic amenities which makes it unconformable for the patients in the settlements.

*Been in the camp for few years, there's no indication of time to leave the camp. Lifestyle in camp is not encouraging* – Person living with leprosy in Taraba State

*Living in a leprosy camp has not been easy, although the people are friendly and our directors are trying, there's a shortage of basic amenities like water, food, and electricity* – Person living with leprosy in Taraba State in Zamfara

*Living here hasn't been easy because we are not being provided with adequate shelter, food, and water*– a person living with leprosy in Taraba State in Zamfara State

Even though workers at the settlements are good towards persons living with leprosy, the living conditions are not palatable.

*The people in the community are good but our condition of living is bad* – Person living with leprosy in Taraba State

*It is difficult for them but we try to improve their welfare and help them resettle after being treated* –Staff of leprosy settlement in Abia

3. Renovating and equipping leprosy settlements

Insufficient government and partner funding for infrastructure construction and maintenance in leprosy settlements is confirmed. Private organizations and NGOs primarily handle maintenance and improvements in living conditions.

*The damages are reported and it is looked into. mostly, private organizations help with renovations and maintenance* – Person living with leprosy in Taraba

*We make fairly decent renovations that will ease their living there and efforts are supported by civil society organizations like Lion's club and Rotary club* – Staff of DAHW

*This settlement is being renovated by the “Daughters of Charity*” – A person living with leprosy in Edo State

4. Stigmatization

Persons living with leprosy are not able to leave the settlement to do business with people outside the settlement because of stigmatization.

*We can't even go out for any business, because people are afraid to buy from us – Person living with leprosy in Niger State. A respondent replied, “ I have been living here for 30 years”. It has not been easy, we face a lot of stigmatization and there are no jobs* – A person living with leprosy in Zamfara

5. Abysmal financing

Study participants expressed concerns about financial challenges affecting business start‐ups and well‐being. The decline in funding significantly impacted their living conditions.

*There's no money to start a business and no money to take care of myself and my family* – Person living with leprosy in Niger State

*We stopped getting proper funding after the Obasanjo regime* – Person living with leprosy in FCT

*The major factor and cause of our lapses here is funding* – Person living with leprosy in Abia state

#### Theme 2: roles organizations play in leprosy control

6. Conducting research, advocacy through media and delivering services

The staff members of DAHW highlight the organization's multifaceted approach to leprosy control. Their activities span capacity building, service delivery and a strong emphasis on research and advocacy.

*We do capacity building and service delivery. We do research, we pride ourselves in the operation of research*. – Staff of DAHW

*We do public awareness and sensitization via traditional and modern media. Another thing we've done is to bring up advocacy to the level in which the rights of persons affected with leprosy are protected in line with the goals of WHO of which Nigeria is a signatory. We also research to gather facts and get clarity on Leprosy control* – Staff of DAHW

7. Programme implementation

TLMN, an NGO operates in 11 states. At the state level, a committee coordinates activities, whereas, at the community level, local committees collect data for funders. TLMN commits funds to support programmes aligned with their mission of leprosy control.

*Currently, we're in about 11 states, As the head of business, my job is to go into the communities, see what the people need and present their needs to funders, get funding, and work with the people to implement. At the state level, we have a project implementation and advisory committee that coordinates the activities. At the community level, we have community management committees formed by the community leader, women leader, and youth leader. We also work with them to get data to send to the funders*. – Staff of TLMN

8. Comprehensive support for leprosy‐affected individuals

Damien Foundation offers empowerment projects, vocational training and employment opportunities to persons living with leprosy. They also provide post‐cure services, educational support and rehabilitated living quarters, including pro bono surgeries for complications.

*We support the NTBLCP in the care and management of persons affected with leprosy. Our area coverage is the southwestern part of Nigeria and Kwara state. Apart from case finding, we also support affected persons with lots of empowerment projects, we also set them up to start a vocation or a trade. Some are even employed to work with Damien Foundation and we pay them* – Staff of Damien Foundation

*Majorly, we offer care after‐cure services. We provide capacity building for health workers, we offer educational support to persons affected with leprosy and their children via the Damien Foundation scholarship scheme, and we support people who are interested through school up to the university/polytechnic level) and the Back To School project, we distribute the usual school pack that involves sandals, school bag, and writing materials to every kid in the settlement, we also provide rehabilitated living quarters for them to enjoy a better living condition, and we also provide pro bono surgeries for people with complications due to leprosy, we have a reconstructive centre in Ogbomosho* – Staff of Damien Foundation

9. Research and health services support

The support rendered by NGOs includes treatment, research, funding for referral centres, reconstructive surgeries, footwear provision and rehabilitative surgeries for leprosy patients.

*It happens in the form of treatment and research. We are providing materials for people infected. We also fund leprosy referral centres and fund reconstructive surgeries*– Staff of DAHW

*We provide appropriate footwear for infected persons, we provide rehabilitative surgeries and make sure they learn how to walk. We also provide care after cure for patients* – Staff of DAHW

*We provide mobile ulcer care project so that we can go into houses of people affected to “dress” their wounds; interestingly, people that ride the bikes and go around are people affected with leprosy* – Staff of Damien Foundation.

Apart from direct medical care to persons living with leprosy, they train healthcare workers to be able to render better services as well.

*We have been training personnel and building the capacity of Nigerian health workers to help them make diagnoses, manage cases, do some reconstructive surgeries, and take care of the social care aspect. We also help with the reintegration of persons affected into society* – Staff of DAHW

#### Theme 3: lessons learned and recommendations

10. Leprosy has become neglected even among neglected diseases

The neglect of leprosy control is evident, compounded by competition for resources, especially with infectious diseases like TB and COVID‐19. NGOs emphasize increased government commitment and funding.

*I will say it's a situation of neglect, not just because the government decides to neglect it but because it's still neglected even among other neglected tropical diseases. It's neglected probably because the government is overwhelmed, there are lots of competing demands in the health budget like COVID‐19 and Tuberculosis. The leprosy control programmes have been joined with the tuberculosis control programme but attention is given more to TB because of how infectious it is*. – Staff of TLMN

*We the NGOs are just here to support, the leprosy control programme is the government's. We demand increased commitment from the state government such that the only form of funding available will not be from the NGOs. The government needs to show more commitment backed up with cash and understand that Leprosy is still a major public health problem*. – Staff of TLMN

#### Summary

Qualitative insights from persons living with leprosy in Nigeria emphasize financial struggles, stigmatization and substandard living conditions in settlements. Limited government funding for infrastructure prompts reliance on private and NGOs. The decline in funding hampers persons living with leprosy's ability to start businesses and provide self‐care. NGOs play pivotal roles in addressing gaps in government support in leprosy care in Nigeria.

## DISCUSSION

The findings from this study showed that the majority of participants were familiar with the signs and symptoms of the disease, whereas just above a tenth revealed a good knowledge level about the cause of leprosy. The low knowledge level of causes might be attributed to a lack of awareness programmes which can be somewhat attributed to the effectiveness of leprosy control initiatives and awareness campaigns. This study showed that most of the participants did not know how leprosy was being transmitted, and this differs from a similar study in Ethiopia where more than half of study participants who are persons living with leprosy knew about leprosy transmission in Ethiopia [16].

The low knowledge level about the cause of leprosy among participants in our study is a critical issue. Participants are familiar with leprosy symptoms but lack adequate knowledge of its cause, emphasizing the need to bridge this awareness gap for effective prevention strategies. It is crucial to emphasize that the low knowledge level regarding the cause of leprosy can have significant public health implications. Individuals with poor knowledge may not take necessary precautionary measures to prevent disease transmission. This finding underscores the need for improved educational programmes and campaigns to address misconceptions and raise awareness about leprosy in Nigeria.

The difference between our study and that of Ethiopia could be attributed to variations in the effectiveness of leprosy control initiatives and awareness campaigns in the respective regions. Poor knowledge of the cause of leprosy and its mode of transmission among persons living with leprosy poses a public health challenge because these individuals would not be exercising the needed precautionary measures to prevent disease transmission.

In this study, the majority of respondents believe that leprosy is treatable. This result tallies with that reported in Ethiopia where respondents believe that leprosy can be treated with pharmaceutical drugs and is in sharp contrast with another study in Mexico where a majority of persons living with leprosy do not know if leprosy is treatable or not [1]. The differences between Ethiopia and similarities with that of Mexico can be a result of various factors, including cultural contexts, methodological variation, socioeconomic factors, historical contexts, awareness campaigns, healthcare infrastructure and variations in study populations. This discrepancy emphasizes the urgency of accurate information dissemination to apprise people of available and efficacious leprosy treatments. Our study emphasizes the need for heightened governmental involvement in leprosy control in Nigeria and we recommend that effort starts with awareness even as the governments start to build more strategic partnerships.

According to our findings, two thirds of participants believed leprosy to be a source of shame, disdain and embarrassment. This is similar to those observed in Thailand and Ethiopia where respondents would deliberately avoid leprosy patients [18, 19].

Although results from our study mentioned that stigmatization has been reduced greatly because of the interventions by NGOs, some persons living with leprosy in this study still mentioned that there is still stigmatization against persons living with leprosy in Nigeria. This was also reported in India in 2019 [20]; where persons living with leprosy mentioned that if they had known, they would not have sought help concerning their condition because of shame and stigma from people in their surrounding environment [20, 21].

This consistent pattern suggests a widespread social stigma attached to leprosy, leading to avoidance behaviours and negative attitudes towards individuals affected by the disease across diverse cultural contexts. The persistence of stigma emphasizes the necessity for continued efforts to combat this issue, which has far‐reaching implications for the mental and social well‐being of those affected by leprosy.

Similar to our findings on stigmatization faced by persons living with leprosy, a systematic review done in 2022 [22] revealed that people living with leprosy are often concerned about their physical appearance which limits their ability to socialize with other people. The study also identified the economic burden of the disease on the patients who cannot move outside the settlements to do business. This supports the findings of our research, where a respondent stated that they cannot freely go out to do business because people are afraid to buy from them. Further, the physical disabilities associated with leprosy and its symptoms limit their ability to work, causing loss of income and unemployment.

Although NGOs have been at the forefront of the fight against leprosy in Nigeria and they have been doing very well as stated in our results, the government needs to take the front row in the fight. One approach to address this is by dividing NTBLCP into three entities, with dedicated units for TB, leprosy and Buruli ulcer. Historically, TB has received more government attention than leprosy, warranting focused efforts on each ailment. This has been reported by Ogbeiwi who indicated that inadequate effort and reporting on leprosy control due to less attention on leprosy compared to TB in recent years by the NTBLCP has shifted the whole burden of care and management of persons with leprosy to ILEP members [23]. Splitting the agency into two enables more oversight, centralized care, improved policy implementation, government funding and coordination at national and sub‐national levels [14]. We also recommend the empowerment of Persons affected with leprosy, increasing the awareness of leprosy, improved intervention from government and organizations and provision of basic amenities by the government.

This study sheds light on the experiences and perceptions of persons living with leprosy in Nigeria. The findings highlight the need for tailored awareness campaigns, accurate information dissemination and government involvement to combat leprosy effectively and address the associated stigma. Comparing our results with studies from other regions underscores the importance of region‐specific strategies in the fight against leprosy and in improving the lives of those affected by the disease.

## CONCLUSION

Overall, this study sheds light on the experience of people living with leprosy in Nigeria, revealing the need for enhanced government involvement, increased funding and improved living conditions for people affected by leprosy. The findings emphasize the importance of comprehensive education, awareness campaigns and the promotion of human rights to counter stigmatization and improve the quality of life for those living with leprosy. Furthermore, the study underscores the evolving approach of reintegration as the best practice, advocating for individuals affected by leprosy to be integrated back into their communities for improved societal inclusion and well‐being. In the face of challenges and opportunities, this study calls for a concerted effort from the government, NGOs and society as a whole to address the multifaceted issues surrounding leprosy control in Nigeria.

## LIMITATION

Despite multiple attempts, we were unable to secure participation from representatives of the NTBLCP and the National Leprosy Eradication Programme (NLEP) within the allocated data collection period. Scheduled interviews were postponed on multiple occasions, and our attempts to conduct virtual interviews were also unsuccessful. Although the selection process was intentionally designed to meet the qualitative objectives of our study, we acknowledge the limitations associated with representativeness and potential bias in the quantitative component. These considerations should be taken into account when interpreting and applying the study findings.

## AUTHOR CONTRIBUTIONS

Gabriel Ilerioluwa Oke, Ifeanyi Nsofor and Bashar Abubakar were involved in the conceptualization and design of the work; Gabriel Ilerioluwa Oke, Don Eliseo Prisno, Ademola Peter Sunday, Ernesto Oluwafemi Dibia, Emmanuel Ebuka Elebesunu and Philip Adewale Adeoye were involved in the acquisition, analysis or interpretation of data for the work. Gabriel Ilerioluwa Oke, Obadiah Okpokpo, Odinaka Kingsley Obeta, Abdulhammed Opeyemi Babatunde, Adeyemi Adebowale Sylvester, Philip Adewale Adeoye and Edith Nnenna Utaka were involved in the drafting of the work and revising it critically for important intellectual content.

## CONFLICT OF INTEREST STATEMENT

Don Eliseo Lucero‐Prisno III is Editor‐in‐Chief of the journal and co‐author of this article and was excluded from the peer‐review process and all editorial decisions related to the acceptance and publication of this article.

## ETHICS STATEMENT

Ethical approval was obtained from the Health Research Ethics Committee of Federal Capital Territory, Nigeria, and consent was obtained as appropriate for all the data collection steps. The anonymity of respondents’ identities was guaranteed. Approval Number: FHREC/2021/01/137/8‐12‐21.

## Data Availability

The data supporting this research are available from the authors on reasonable request.
